# Dynamics of HEV viremia, fecal shedding and its relationship with transaminases and antibody response in patients with sporadic acute hepatitis E

**DOI:** 10.1186/1743-422X-7-213

**Published:** 2010-09-06

**Authors:** Nidhi S Chandra, Asha Sharma, Bharti Malhotra, Ramesh R Rai

**Affiliations:** 1 Department of Gastroenterology, SMS Medical College and Hospital, Jaipur (Rajasthan), India; 2 Department of Zoology, University of Rajasthan, Jaipur, India; 3 Department of Microbiology, SMS Medical College, Jaipur (Rajasthan), India

## Abstract

**Background:**

There is paucity of data regarding duration of fecal excretion and viremia on sequential samples from individual patients and its correlation with serum transaminases and antibody responses in patients with acute hepatitis E. This prospective study was undertaken at a tertiary care center in Northern India over 15 months. Only those patients of sporadic acute hepatitis E who were in their first week of illness and followed up weekly for liver function tests, IgM anti HEV antibody and HEV RNA in sera and stool were included. HEV RNA was done by RT - nPCR using two pairs of primers from RdRp region of ORF 1 of the HEV genome.

**Results:**

Over a period of 15 months 60 patients met the inclusion criterion and were enrolled for the final analysis. The mean age of the patients was 29.2 ± 8.92 years, there were 39 males. The positivity of IgM anti HEV was 80% at diagnosis and 18.3% at 7th week, HEV RNA 85% at diagnosis and 6.6% at 7th week and fecal RNA 70% at the time of diagnosis and 20% at 4th week. The maximum duration of viremia detected was 42 days and fecal viral shedding was 28 days after the onset of illness.

**Conclusion:**

Present study reported HEV RNA positivity in sera after normalization of transaminases. Fecal shedding was not seen beyond normalization of transaminases. However, viremia lasted beyond normalization of transaminases suggesting that liver injury is independent of viral replication.

## Background

Hepatitis E virus is the etiological agent of non-HAV enterically transmitted hepatitis and major cause of sporadic as well as epidemic hepatitis [1,2]. In Indian subcontinent, it accounts for 30-60% of sporadic hepatitis [3,4]. One distinct feature of hepatitis E, compared with other forms of viral hepatitis is its higher incidence and severity in pregnant woman [5]. The overall mortality rate of hepatitis E is generally lower than 1% but it can be as high as 20-25% among pregnant women [6].

Being a disease of developing countries a fair amount of information has been generated from India. There is paucity of data regarding duration of fecal excretion and viremia on sequential multiple samples from individual patients and its relationship with serum transaminases and IgM antibody response. This information is vital for understanding pathogenesis and transmission dynamics of acute hepatitis E. The information is either from a human volunteer who ingested HEV [7] or a study [8], based predominantly on pooled data of single sample from different patients during HEV epidemics. Data on sequential samples obtained from individual patients is scant.

Two studies with relatively less number of patients have looked for viremia and fecal shedding at varying but not at regular intervals, the samples were collected as and when the patients attended the clinics but not at a fixed schedule [9,10]. Only in a recent Chinese study, small number of patients (n = 32) were tested for viremia in a sequential manner but fecal shedding and IgM and anti-HEV were not studied [11]. The present study has been undertaken where patients with sporadic acute viral hepatitis were prospectively evaluated for transaminases, HEV viremia, HEV fecal shedding, and IgM antibody in multiple series samples obtained from individual patients at weekly interval. Also, these parameters of acute hepatitis E were compared between pregnant and non-pregnant females.

## Materials and methods

### Study population

The present study was undertaken prospectively at a tertiary care center in Rajasthan, India. The study period extended from 1^st ^Jan 2007 to 31^st ^Jan 2008 over 13 months. The study was approved by the institutional ethics committee and informed written consent was taken from the patients. The diagnosis of acute hepatitis E was made on the basis of clinical presentation, raised transaminases and Bilirubin, and positive IgM anti HEV antibody and/or HEV RNA in sera. Only those patients of sporadic acute hepatitis E who were in their first week of illness, followed up weekly for liver function tests, IgM anti HEV antibody and HEV RNA for final analysis and those were failing these criteria excluded from the study. Patients with concomitant positive IgM anti HAV, IgM anti HBc or anti HCV (i.e. dual infection) and patients with underlying alcoholic liver disease were also excluded from the study.

### Sample Collection and Handling

The patients were asked to follow up weekly intervals after the first visit. At each visit clinical sign and symptoms were noted. All events were measured with reference to day of the first symptoms. Serum and stool samples were collected, coded and stored at -80°C till processing. The stool and serum samples were obtained for subsequent two weeks after the clearance of virus from serum and stool to avoid any error and confirm the negativity.

Biochemical analyses that include serum Bilirubin, ALT, AST and serum alkaline phosphatase was done at each visit by automated analyzer in the central laboratory of the institute. Coded sera of patients and positive and negative controls were tested for IgM anti- HEV using commercially available kit (Globe diagnostic SRL, Italy).

### RT-PCR

**Figure 1:**
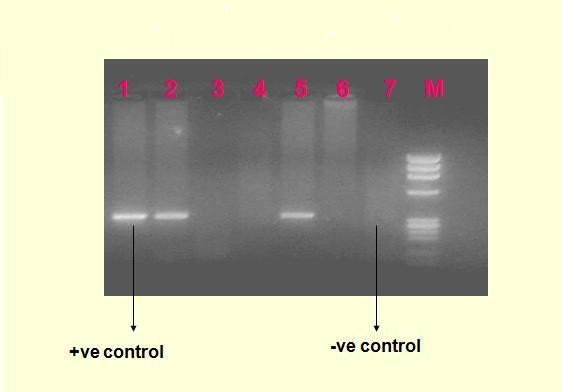
**Agarose gel electrophoresis shows HEV specific 343 base pair amplified product M**: 100 bp ladder of molecular weight markers; lanes 1: positive control; lanes 2-6: five serum specimens from patients with hepatitis E; lane 7: negative control from saline.

Extracted RNA by GITC chloroform phenol method with minor modification [12] was subjected for cDNA synthesis. cDNA synthesis was carried out using MuLV RT enzyme, reverse primer (20 pmol/ml), RNase out (20 U/μl, Gibco BRL), 0.1 M DTT and 5 μl templates at 42°C for one hour. After cDNA synthesis PCR amplification was carried out using the specific primers selected from nonstructural ORF-1 region (Gene Bank accession no. M-32400) [1]. The thermal cycling conditions were initial denaturation 94°C for 5 minutes followed by 30 cycles of denaturation for 30 seconds at 94°C, annealing for 30 seconds at 59°C and extension for 30 seconds at 72°C, as well as final extension for 7 minutes at 72°C. The final PCR products were checked out on 2% gel electrophoresis stained with ethidium bromide (10 mg/ml) under UV transillminator. Figure 1 depicts the agarose gel electrophoresis of HEV specific 343 base pair amplified product.

### Statistical Analysis

For data management and statistical analysis, SPSS-10 software (SPSS Inc., Chicago, IL)was used. Baseline laboratory markers were expressed as mean values with standard deviation. Difference between pregnant and non-pregnant females with respect to various liver function tests, and duration of persistence of IgM anti-HEV, HEV viremia and HEV fecal RNA was calculated using the student t-test. P value of less than 0.05 was considered significant.

## Results

Over a period of thirteen months there were 60 patients met the inclusion criterion and were enrolled for the final analysis rest were excluded from the study because of lack of desired follow up. The mean age of the patients was 29.2 ± 8.92 years (range: 11 to 54 years) and there were 39 males and 21 females; there were 10 pregnant and 11 non-pregnant females. Five pregnant females were in third trimester, four in second trimester and one in first trimester. Three pregnant females developed acute liver failure, 3 developed miscarriages and 1 died (after completion of study in the 3^rd ^month of illness), all pregnant females with complicated diseases were in the third trimester. Uncomplicated acute hepatitis E was seen in 44 patients and acute liver failure in 16 patients.

335 sera samples were studied for HEV RNA and 212 stool samples were studied for fecal RNA. The mean levels of serum Bilirubin, AST, ALT and alkaline phosphate and their fall over days are shown in the table 1.

**Table 1:** Weekly levels of various liver function tests

Days	S. Bilirubin(mg/dL)	S. AST(U/L)	S. ALT(U/L)	SAP(U/L)
**0-7**	7.02 ± 3.5	961.5 ± 575.8	1145.1 ± 773	773 ± 643.5

**8-14**	6.69 ± 3.32	750 ± 717.6	746.1 ± 677.1	599.8 ± 521

**15-21**	5.97 ± 5.34	153.9 ± 130.4	253.3 ± 237.6	424.2 ± 265.8

**22-28**	4.91 ± 4.37	111.8 ± 67.67	147.2 ± 119	444.8 ± 346.5

**29-35**	2.98 ± 2.76	56.17 ± 30	73.47 ± 69	333.3 ± 318.8

**36-42**	1.2 ± 1.03	43.5 ± 16.3	45.5 ± 18	298.7 ± 276.37

***37-49***	0.96 ± 0.64	40.1 ± 21.08	40.8 ± 10.8	271.4 ± 235.6

Data expressed as mean ± SD

### Diagnosis of acute hepatitis E

There were thirteen patients with negative IgM anti HEV but positive HEV RNA. There were eight patients with positive IgM anti HEV and negative HEV RNA at presentation; rest 39 patients had positivity of both IgM anti HEV and HEV RNA.

### Positivity of various markers of HEV infection

**Figure 2:**
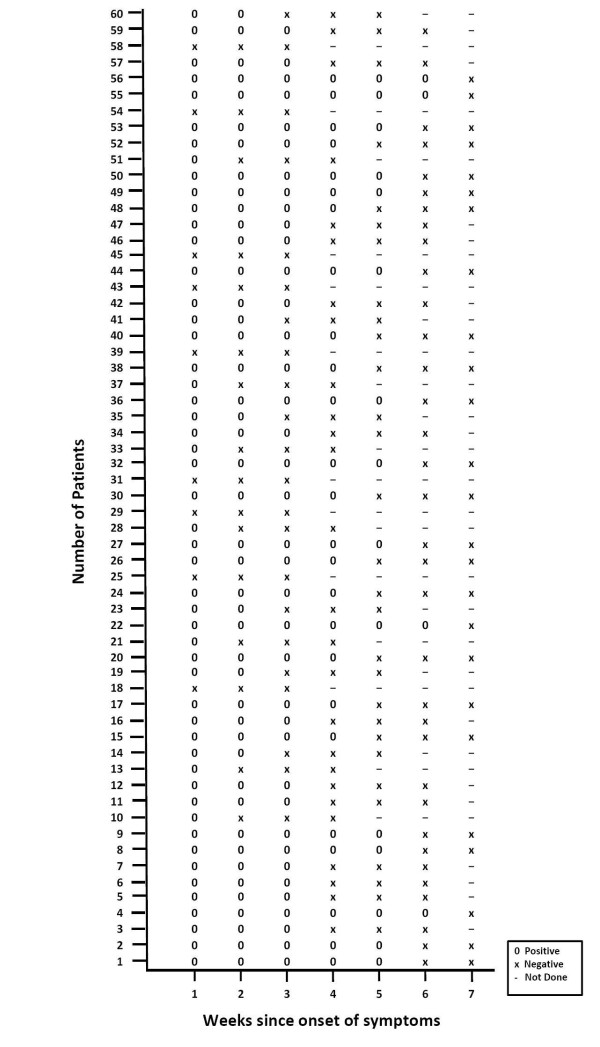
**HEV RNA in sera of 60 patients done at weekly interval**.

**Figure 3:**
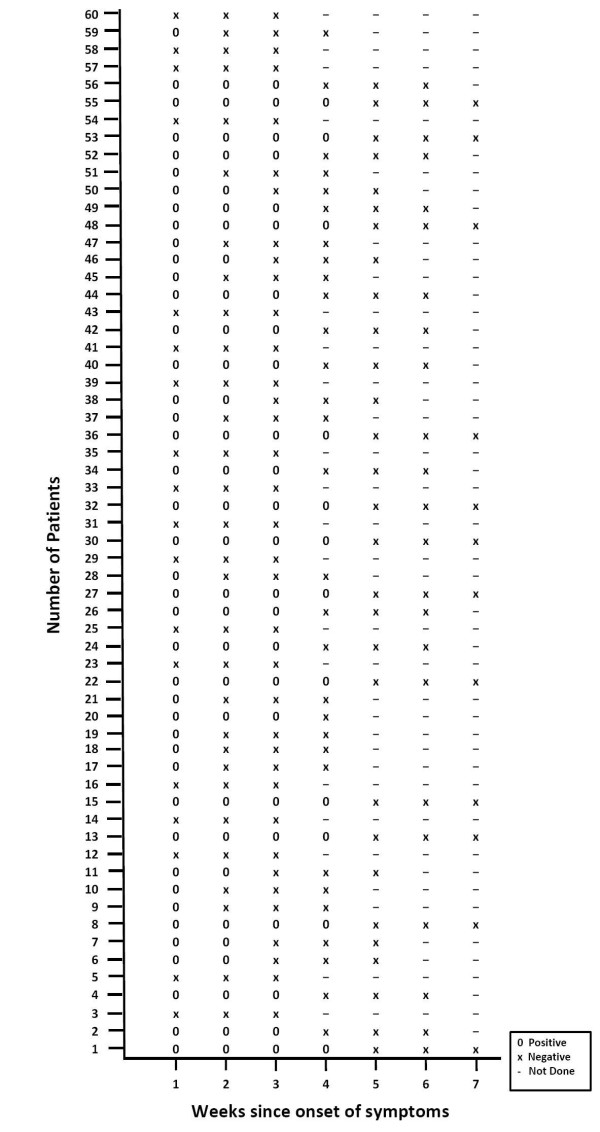
**HEV RNA in stool of 60 patients done at weekly interval**.

The positivity of IgM anti HEV was 78.3% at diagnosis and 18.3% at 7^th ^week, HEV RNA 86.7% at diagnosis and 6.6% at 6^th ^week and fecal shedding of HEV RNA 70% at the time of diagnosis and 20% at 4^th ^week and in 5^th ^week all the samples were negative. Table 2 shows the positivity of IgM anti HEV, serum HEV RNA and fecal viral shedding over weeks. The first to disappear is fecal shedding followed by HEV RNA and then IgM anti HEV. The maximum duration of HEV viremia was 42 days, HEV fecal shedding 28 days and IgM anti HEV 49 days. Protracted viremia i.e. persistence of HEV RNA beyond normalization of ALT was seen in 4 patients up till day 42. Figure 2 and 3 show the results of HEV viremia and fecal shedding respectively in all 60 patients followed weekly over an interval of 7 weeks. If any of the test was negative at a particular week, two more samples were tested in subsequence two weeks to confirm negativity.

**Table 2:** Positivity of IgM anti HEV, HEV RNA and fecal viral shedding in serial samples of patients with acute sporadic hepatitis E (N = 60)

**Days**^**†**^	IgM anti HEV	HEV RNA in sera	Fecal Viral shedding
**1-7**	48(78.3)	51(86.7)	42(70)

**8-14**	43(71.6)	44(73.3)	30(50)

**15-21**	40(66.6)	38(63.3)	24(40)

**22-28**	35(58.3)	25(41.6)	12(20)

**29-35**	30(50)	15(25)	0(0)

**36-42**	26(43.3)	4(6.6)	0(0)

***43-49***	11(18.3)	0(0)	0(0)

Data expressed as number (percentage) and Days after the onset of illness

Mean ALT activity was higher in sera collected 1-7 days and declined there after. This suggested that liver injury is highest during initial stages of infection. HEV RNA was detected in 86.7% sera collected during first seven days of illness when the ALT level was maximum.

### Clinical parameter and pregnancy

The maximum duration of viremia was 42 days and 36 days, fecal shedding 28 days and 19 days and IgM anti HEV 46 days and 49 days in pregnant females and non-pregnant patients respectively. Table 3 shows the comparison of liver function tests, duration of persistence of IgM anti HEV, HEV viremia and fecal HEV shedding between pregnant and non-pregnant females. The two group did not differ significantly except for fecal shedding (P = 0.006), viremia (P = 0.016) and mortality rate(P = 0.010) that was significantly higher in pregnant females.

**Table 3:** Comparison of various liver function tests and persistence of various markers of HEV infection in pregnant and non-pregnant females

	Pregnant females (N = 10)	Non-Pregnant females (N = 11)	P value
S. bilirubin(mg %)	8.84 ± 2.23	7.518 ± 4.72	0.44

S. AST (IU)	1156.4 ± 183.04	925.81 ± 527.86	0.20

S. ALT (IU)	1295.3 ± 877.95	1272.53 ± 831.88	0.95

S. ALP (IU)	956.6 ± 511.51	1103 ± 814.77	0.63

Viremia^†^	36.1 ± 5.82	29.09 ± 5.87	0.016

Fecal shedding^†^	18.4 ± 4.19	13.0 ± 3.72	0.006

IgM anti HEV^†^	40.1 ± 5.80	39.90 ± 4.86	0.84

Mortality (no.)	2	0	

All values given in mean ± SD

^† ^Maximum duration of persistence in days

## Discussion

Hepatitis E is an important etiological agent of epidemic and sporadic hepatitis associated with high morbidity and mortality in pregnant females. The pathogenesis and rate of transmission of hepatitis is not very clear. Information on the duration of IgM anti HEV, viremia and fecal shedding is very important to understand the transmission dynamics and pathogenesis of hepatitis E, but related data are particularly limited.

Therefore, the present study explained IgM anti HEV, viremia, fecal shedding and level of transminases in individual patient with acute sporadic hepatitis E. we were selected 60 patients who provided sequential samples for the study. The serial samples obtained from the individual patients were studied for IgM anti HEV and HEV RNA in sera and fecal matter weekly till disappearance of HEV. Two subsequent samples were tested to confirm persistent negativity for HEV RNA. This is in comparison to a recent study from China that studied serial samples (at around 5 day interval) in patients with acute hepatitis E and looked for HEV viremia [11]. A study explained 26 patients with acute sporadic hepatitis E, the samples were collected as and when the patient came in contact with the author's for IgM anti HEV, HEV viremia and fecal shedding [10]. Similar method of sample collection was reported in another study [9]. Few studies based on a volunteer and single sample from patients are also available [7,8].

In the present study diagnosis of acute hepatitis E was based on either IgM anti HEV or HEV-RNA positivity. Positivity of IgM anti HEV (78.3%) was less than the positivity for HEV-RNA (86.7%) thus indicating that HEV RNA may be slightly better indicator for ongoing HEV infection and hence its diagnosis. Even though HEV RNA was better than anti HEV for diagnosis of acute hepatitis, it cannot be a better test in routine, as RT-PCR is cumbersome and costly. However, in a setting of acute hepatitis if routine viral markers are negative, HEV RT-PCR may be the next useful tool of investigation.

In the present study 13/60 (21.6%) patients were positive for HEV- RNA but negative from week 1 onwards. Possible explanation for its negativity could be i.) low sensitivity of the ELISA test used [13] ii.) sequence variation among different genotypes [14] and iii.) a poor host immune response to HEV infection [15]. Some patients were positive for IgM anti HEV but negative for HEV-RNA (13.3%), reason behind that the viremia is short lived and disappeared prior to development of icterus or early in 1^st ^week of development of icterus and the variation of nucleotide sequence in the primary regions among different HEV strains could be as high as 28%, which may account for the difficulty in PCR amplification [14].

In present series IgM anti HEV, viremia and fecal shedding could be detected up to 49 days, 42 days and 28 days respectively after onset of illness. We have shown earlier IgM anti HEV was positive up to 45 days [16] and in another Indian study IgM anti HEV was positive up to 21-112 days after iceterus [10]. A study from China on serial sample in 32 patients viremia was detected till 35 days after the onset of illness and in other group of randomly selected samples maximum duration of viremia was noted 51 days after the onset of illness [11]. In another study viremia was lasted for a maximum period of 45 days after the onset of illness [9]. In a human self inoculation study with hepatitis E virus, viremia was detected to last for 16 days [7]. In another were single serum samples from patients with acute hepatitis E was collected, HEV-RNA was detected in 7% and 91% of serum sample collected on days 0-3 and 8-11 respectively; however, only two of the 11 serum samples obtained during days 27-41 tested positive [8]. In an Indian study, fecal shedding was studied in only 4 patients, at varying intervals and lasted 9, 10, 12 and 52 days [10]. Fecal shedding was detected less frequently than viremia. This finding is similar to earlier reports [8,10,17]. The reason for this remains unknown. Therefore, the present and previous reports suggest that detection of fecal viral shedding is a less desirable event for diagnostic approach than detection of viremia.

Till now there have been limited data including the present study that talks of protracted viremia. The concept of protracted viremia was first given by Nanda et al and reported four patients in whom viremia extended beyond the normalization of ALT; they concluded that these patients may act as short term reservoirs for propagation of sporadic hepatitis E [10]. However, how they act as reservoir was not mentioned. Aggarwal R et al reported 1 case as protracted viremia and there were 4 such cases in the present study [9]. Viremia that lasts beyond normalization of transaminases may suggest that liver injury is independent of viral replication. The exact importance of this concept is not known.

In the present study the duration of viremia, fecal shedding and mortality was significantly higher in pregnant females in comparison to non-pregnant females but duration of persistence of IgM antibodies and other liver function tests were not different. This data is not available in the literature to the best of our literature search. However, the number of patients in both the groups was small and would need further research on large number of patients to reach any statistical conclusion. Whether the pregnant females contribute more to maintain the pool of hepatitis E in the society also needs further studies. The authors feel that viral load may be an important factor determining the outcome of acute hepatitis E in pregnancy as has been shown by a recent Indian study [18].

## Conclusion

This is the largest study that analyzed 60 patients of acute sporadic hepatitis E prospectively for IgM anti HEV, viremia and fecal shedding. HEV RNA was better than IgM anti HEV for diagnosis of acute hepatitis; still its routine use for diagnosis of acute hepatitis E is not feasible except in patients with negative IgM anti HEV, high level of suspicion and in research setting. It was observed that viremia lasts for a longer period than fecal shedding in most patients. Although fecal shedding was not seen beyond normalization of transaminases. Viremia lasted beyond normalization of transaminases in some patients and this may suggest that liver injury is independent of viral replication. Viremia and fecal shedding did not last too long to be responsible for maintenance of HEV virus in the environment. The present study also provides data on pregnant females for the first time and duration of viremia and fecal shedding was significantly more than non-pregnant females.

## Competing interests

The authors declare that they have no competing interests.

## Authors' contributions

NS performed most experiments related to the study like ELISA, RNA extraction, RT - nPCR and wrote the manuscript. RR provided clinical samples from the HEV infected patients for the study, helped in editing the manuscript. Some help was given by BM and AS in the design of the study. All authors read and approved the final manuscript.
